# A structured narrative review of clinical and experimental studies of the use of different positive end‐expiratory pressure levels during thoracic surgery

**DOI:** 10.1111/crj.13545

**Published:** 2022-10-01

**Authors:** Jiang Yueyi, Tan Jing, Gu Lianbing

**Affiliations:** ^1^ The Affiliated Cancer Hospital of Nanjing Medical University Nanjing China; ^2^ Department of Anesthesiology Jiangsu Cancer Hospital Nanjing China

**Keywords:** lung protective ventilation, one‐lung ventilation, positive end‐expiratory pressure, thoracic

## Abstract

**Objectives:**

This study aimed to present a review on the general effects of different positive end‐expiratory pressure (PEEP) levels during thoracic surgery by qualitatively categorizing the effects into detrimental, beneficial, and inconclusive.

**Data source:**

Literature search of Pubmed, CNKI, and Wanfang was made to find relative articles about PEEP levels during thoracic surgery. We used the following keywords as one‐lung ventilation, PEEP, and thoracic surgery.

**Results:**

We divide the non‐individualized PEEP value into five grades, that is, less than 5, 5, 5–10, 10, and more than 10 cmH_2_O, among which 5 cmH_2_O is the most commonly used in clinic at present to maintain alveolar dilatation and reduce the shunt fraction and the occurrence of atelectasis, whereas individualized PEEP, adjusted by test titration or imaging method to adapt to patients' personal characteristics, can effectively ameliorate intraoperative oxygenation and obtain optimal pulmonary compliance and better indexes relating to respiratory mechanics.

**Conclusions:**

Available data suggest that PEEP might play an important role in one‐lung ventilation, the understanding of which will help in exploring a simple and economical method to set the appropriate PEEP level.

## Graphical abstract

General effects of different positive end‐expiratory pressure (PEEP) levels during thoracic surgery are summarized. We divide the non‐individualized PEEP value into five grades, that is, less than 5, 5, 5–10, 10, and more than 10 cmH_2_O, among which 5 cmH_2_O is the most commonly used in clinic at present. Individualized PEEP is adjusted by test titration or imaging method to adapt to patients' personal characteristics.

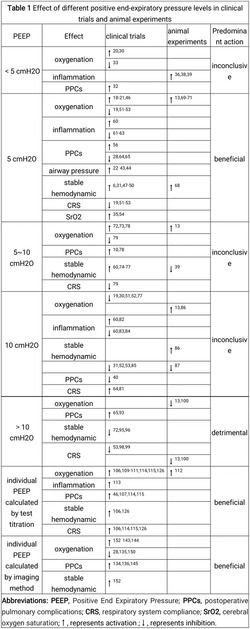

## 1 INTRODUCTION

Positive end‐expiratory pressure (PEEP) is an auxiliary ventilation mode in which the airway pressure and alveolar pressure are higher than the atmospheric pressure through ventilator intervention in the expiratory phase. Applying a certain level of PEEP to the ventilated lung can promote lung recruitment, provide sufficient oxygenation and alveolar ventilation, increase functional residual capacity (FRC), reduce intrapulmonary shunt, and effectively improve alveolar compliance.^1^, ^2^, ^3^, ^4^, ^5^, ^6^

One lung ventilation (OLV) realizes lung isolation and provides a good surgical field for surgical operation. However, OLV is a non‐physiological ventilation mode, and unreasonable respiratory parameters often lead to ventilator‐reduced lung injury.^7^ With the gradual improvement of the concept of lung‐protective ventilation strategy (LPVs), LPVs is increasingly being used in thoracic surgery low tidal volume (VT) ventilation mode, PEEP, and recruitment maneuver strategy (RMS) are common strategies used in clinical practice.^8^, ^9^, ^10^ Currently, the concept of low tidal volume ventilation has been accepted and widely used. Although low tidal volume ventilation can reduce the risk of hyperinflation, the cyclic opening and closing of alveoli reduce the stability of alveoli, which may further lead to atelectasis.^11^, ^12^, ^13^, ^14^ Therefore, the application of PEEP appears to be protective. It can dilate the collapsed alveoli during surgery and prevent the expanded alveoli from collapsing again throughout the respiratory cycle.^15^, ^16^, ^17^ It also plays a certain role in improving ventilation and oxygenation. Nevertheless, how to set the appropriate level of PEEP remains questionable and controversial even after a large number of animal experiments and clinical trials.

Most scholars adopted that PEEP at the level of 5 cmH_2_O is enough to keep the alveoli open, improve the oxygenation function of lung tissue, and reduce the shunt fraction.^18^, ^19^, ^20^, ^21^ On the other hand, some scholars argued that it may not lead to excessive alveolar expansion and hemodynamic damage.^22^ They believed that 10 cmH_2_O PEEP can significantly increase the FRC, arterial oxygen partial pressure, and reduce the dead space fraction.^23^ Besides this, there exists several other points of views. More recently, the concept of optimal PEEP level has been put forward for it can improve intraoperative oxygenation and reduce the incidence of perioperative respiratory complications.^24^, ^25^, ^26^, ^27^ To determine an optimal PEEP level that minimizes the harm caused by either insufficient or excessive distension, an individual approach may be needed.^28^ Overall, definitive evidence on the optimal PEEP value is not yet available. The review compared not only different PEEP levels but also possible optimal strategies during thoracic surgery.

## 2 SEARCH STRATEGY

Electronic databases were searched until March 2022 using a string of medical subject heading terms or related terms. The sensitive search strategy combined the following Medical Subject Headings and Keywords: “one‐lung ventilation” OR “OLV” AND “positive end–expiratory pressure” OR “positive end expiratory pressure” OR “PEEP” AND “thoracic surgery.” Pubmed was used as a search engine for the Medline database and as the main source of information for this review. Furthermore, the China National Knowledge Infrastructure (CNKI) and Wanfang Database were also searched for relevant articles. The language was restricted to English and Chinese. The article type included clinical trials, animal experiments, observational studies, and review articles. In addition, we searched related articles in the reference lists of the included articles. Selection of clinical trials was restricted to those adult patients selective for thoracic surgery undergoing general anesthesia. Clinical trials in patients undergoing cardiac surgery and lung transplantation were excluded. A total of 160 articles were selected and analyzed in this review. Then, three authors reviewed these retrieved articles for suitability.

This narrative review provides a comprehensive literature of clinical trials and animal experiments that involved using PEEP in thoracic surgery, the application of which has its own advantages and disadvantages. When searching electronic databases, we found that a mass of clinical or animal research applied PEEP value of 5 or 10 cmH_2_O, with the remainder in between or less than 5 cmH_2_O or more than 10 cmH_2_O. The studies have come to different conclusions even though the same PEEP value used. Thus, we not only divided the non‐individualized PEEP value into five grades, that is, less than 5, 5, 5–10, 10, and more than 10 cmH_2_O, divided the individualized PEEP value into two main setting approaches, test titration method and imaging method, but also qualitatively categorized the conclusions from the papers into detrimental, beneficial, and inconclusive. General effects of different PEEP levels during thoracic surgery are summarized in Table 1.

**TABLE 1:** Effect of different positive end‐expiratory pressure levels in clinical trials and animal experiments

PEEP	Effect	Clinical trials	Animal experiments	Predominant action
<5 cmH_2_O	Oxygenation	↑^20^, ^30^		Inconclusive
		↓^33^		
	Inflammation		↑^36^, ^38^, ^39^	
	PPCs	↑^32^		
5 cmH_2_O	Oxygenation	↑^18^, ^19^, ^20^, ^21^, ^46^	↑^13^, ^69^, ^70^, ^71^	Beneficial
		↓^19^, ^51^, ^52^, ^53^		
	Inflammation	↑^60^		
		↓^61^, ^62^, ^63^		
	PPCs	↑^56^		
		↓^28^, ^64^, ^65^		
	Airway pressure	↑^22^, ^43^, ^44^		
	Stable hemodynamic	↑^6^, ^31^, ^47^, ^48^, ^49^, ^50^	↑^68^	
	CRS	↓^19^, ^51^, ^52^, ^53^		
	SrO_2_	↑^35^, ^54^		
5–10 cmH_2_O	Oxygenation	↑^72^, ^73^, ^78^, ^80^	↑^13^	Inconclusive
		↓^81^		
	PPCs	↑^10^, ^78^		
	Stable hemodynamic	↑^60^, ^74^, ^75^, ^76^, ^77^	↓^39^	
	CRS	↑^80^		
		↓^81^		
10 cmH_2_O	Oxygenation	↑^64^	↑^13^, ^88^	Inconclusive
		↓^19^, ^30^, ^51^, ^52^, ^77^		
	Inflammation	↑^60^, ^84^		
		↓^60^, ^85^, ^86^		
	Stable hemodynamic		↑^88^	
		↓^31^, ^52^, ^53^, ^87^	↓^89^	
	PPCs	↓^40^		
	CRS	↑^64^, ^83^		
>10 cmH_2_O	Oxygenation		↓^13^, ^102^	Detrimental
	PPCs	↑^65^, ^95^		
	Stable hemodynamic	↓^72^, ^97^, ^98^		
	CRS	↓^53^, ^100^, ^101^	↓^13^, ^102^	
Individual PEEP calculated by test titration	Oxygenation	↑^108^, ^112^, ^113^, ^114^, ^118^, ^119^, ^127^, ^132^, ^138^	↑^116^	Beneficial
	Inflammation	↑^117^		
	PPCs	↑^46^, ^109^, ^118^, ^119^		
	Stable hemodynamic	↑^108^, ^132^, ^138^		
	CRS	↑^108^, ^115^, ^118^, ^119^, ^127^, ^132^, ^138^		
Individual PEEP calculated by imaging method	Oxygenation	↑^151^, ^152^, ^160^		Beneficial
		↓^28^, ^143^, ^158^		
	PPCs	↑^142^, ^144^, ^153^		
	Stable hemodynamic	↑^160^		

Abbreviations: CRS, respiratory system compliance; PEEP, positive end‐expiratory pressure; PPCs, postoperative pulmonary complications; SrO_2_, cerebral oxygen saturation; ↑, represents activation; ↓, represents inhibition.

## 3 NON‐INDIVIDUALIZED PEEP VALUE

### 3.1 PEEP < 5 cmH_2_O

The precise role of low‐level PEEP has been less clearly defined. PEEP less than 5 cmH_2_O is rarely used in clinical practice, possibly because it cannot fully expand the alveoli or resist the patient's intrinsic PEEP.^29^

#### 3.1.1 Clinical research

In the Department of Thoracic Surgery, one‐lung ventilation is widely used during operations. During one‐lung ventilation, partial pressure of oxygen may decrease and peak airway pressure may increase. The application of PEEP levels of less than or equal to 5 cmH_2_O can improve oxygenation and to correct hypoxemia,^20^, ^30^ while avoiding high airway pressure and lung injury caused by excessive alveolar expansion,^31^ thereby reducing the incidence of postoperative pulmonary complications and facilitating the recovery of respiratory function.^32^

Nevertheless, a recent meta‐analysis investigating the association between intraoperative PEEP settings and outcomes suggested that there were no significant differences between “low” PEEP (3–5 cmH_2_O) and “high” PEEP (8–10 cmH_2_O) in improving the intraoperative partial pressure of oxygen and the incidence of postoperative pulmonary complications,^33^ but lower incidence of intraoperative hypotension in the low‐level PEEP group. The reason is that despite increasing the intrathoracic pressure, low‐level PEEP may not affect venous return and cardiac filling Blank et al. have found that low levels of PEEP during OLV are associated with increased postoperative pulmonary complications and a higher mortality.^34^ Current evidence is insufficient to show the advantage of low PEEP. A randomized controlled trial, which compares high with low PEEP in patients receiving one‐lung ventilation for thoracic surgery is going on.^35^ Further high‐quality randomized controlled trials are needed to establish the clinical effect of low‐level PEEP. When the results come out, we will further deepen our understanding.

#### 3.1.2 Animal research

In the rabbit lung model in vitro, Steinberg et al. found that 3 cmH_2_O PEEP could maintain the stability of alveoli, significantly reduce the level of inflammatory cytokines (IL‐6),^36^ and cause less damage to lung tissue than 10 cmH_2_O PEEP.^37^ Another study showed that 1 cmH_2_O PEEP had lower levels of inflammatory cytokines associated with lung injury than 0 cmH_2_O PEEP.^38^ Naikas et al. performed one lung ventilation on lambs with different levels of PEEP. The results showed that compared with 0 and 7 cmH_2_O PEEP, 4 cmH_2_O PEEP could lower protein leakage, reduced mRNA expression of neutrophils and proinflammatory cytokines, which could minimize the pro‐inflammatory response caused by ventilation and protect the lung from injury.^39^

### 3.2 5 cmH_2_O PEEP

Currently, 5 cmH_2_O PEEP has achieved much recognition through animal experiments and a large range of clinical studies. They believed that 5 cmH_2_O PEEP is enough to keep the alveoli open during OLV with not only improve the oxygenation function of lung tissue, reduce shunt fraction, ameliorate ventilation perfusion, and prevent hypoxemia^18^, ^19^, ^20^, ^21^ but not lead to excessive expansion of alveoli and increase of airway pressure.^22^, ^40^

#### 3.2.1 Clinical research

The 5 cmH_2_O PEEP has been widely used during one lung ventilation in clinic.^34^, ^41^, ^42^ Based on their findings, the investigators consider that 5 cmH_2_O PEEP is safe with a trend of reducing the peak airway pressure, plateau airway pressure, driving pressure,^43^, ^44^ and making the gas in the lung more evenly distributed.^45^ Peter D. Slinger et al. applied 5 cmH_2_O PEEP during one lung ventilation and then found the platform end‐expiratory pressure moved to the inflection point of the static compliance curve, which may contribute to the improvement of oxygenation.^46^

Hoftman N et al. evaluated the role of 5 cmH_2_O PEEP on hemodynamics during OLV. Except for a slight decrease in stroke volume and cardiac output per minute, pulmonary artery pressure, mean arterial pressure, mixed venous oxygen saturation, and left ventricular ejection time had no significant changes.^6^, ^31^, ^47^, ^48^, ^49^, ^50^ In a study involving 60 patients undergoing thoracoscopic bullectomy surgery, Li Wei et al. demonstrated no significant difference in the improvements in pulmonary dynamic compliance and arterial oxygenation between the 5 cmH_2_O PEEP and 10 cmH_2_O PEEP study groups by observing the changes of blood gas analysis and hemodynamics.^19^, ^51^, ^52^, ^53^ And yet, 5 cmH_2_O PEEP appeared to improve cerebral perfusion, cerebral oxygenation, and reduce the decline of cerebral blood oxygen saturation,^35^, ^54^ which is helpful to ameliorate postoperative brain function, reduce the incidence of postoperative delirium,^55^ postoperative pulmonary complications, and total complications. Consequently, it can be seen that the role of 5 cmH_2_O PEEP cannot be overemphasized in reducing the risk of ventilator‐induced lung injury and postoperative respiratory complications,^56^ and contributing to the rapid postoperative recovery of patients.^11^, ^43^, ^44^, ^57^, ^58^

Otherwise, a few studies were reported the influence of 5 cmH_2_O PEEP on immunization. By monitoring surrogate markers of immunization, 5 cmH_2_O PEEP could reduce NK cells, CD3^+^, CD4^+^ levels after operation, which may reduce the inhibition of postoperative cellular immune function.^59^

Meanwhile, whether 5 cmH_2_O PEEP is effective in alleviating inflammatory response is currently unknown. The investigators compared ZEEP versus 5 cmH_2_O PEEP in patients undergoing esophageal surgery; they found significantly lower serum makers of inflammation (IL‐1b, IL‐6, and IL‐8) in the 5 cmH_2_O PEEP group.^60^ The systemic pro‐inflammatory response weakened, and the damage to alveolar epithelial cells reduced thereby. Nevertheless, other studies demonstrated that there were no major differences in carbon monoxide, interleukin‐6, and malondialdehyde.^61^, ^62^, ^63^ The 5 cmH_2_O PEEP does not appear to be beneficial in terms of oxidative stress, inflammatory response, or respiratory complications.^63^

When PEEP is 5 cmH_2_O, FRC can be increased by 500 ml. Theoretically, it can reduce the incidence of atelectasis caused by ventilation‐perfusion (V/Q) mismatch and hypoxemia. On the contrary, some scholars have indicated that 5 cmH_2_O PEEP is not enough to keep the alveoli opening and prevent the occurrence of atelectasis.^28^, ^64^ According to the study of Cylwik J et al., 87% of patients treated with 5 cmH_2_O PEEP have subpleural atelectasis.^65^ The presence of atelectasis has the potential to cause inflammation and alveolar injury, temporarily increase extravascular lung water, and induce pulmonary oedema, thus increasing the risk for pneumonia.^66^ The 5 cmH_2_O PEEP applied during thoracic surgery is hemodynamically well tolerated; however, it does not improve oxygenation in all cases.

From the existing evidence, 5 cmH_2_O PEEP whose advantages outweigh disadvantages has many benefits such as dilate collapsed alveoli, maintain relatively normal end respiratory lung volume, ameliorate pulmonary gas diffusion, and correct hypoxia.

#### 3.2.2 Animal research

In a sheep model of pneumonectomy, a tidal volume of 6 ml/kg with 5 cmH_2_O PEEP did not increase significantly in extravascular lung water compared with ZEEP groups.^67^ Michelet et al. investigated the variation of oxygenation at different levels of PEEP in pigs with left‐lung ventilation. The 5 cmH_2_O PEEP was pertinent to improving oxygenation and continuous lung recruitment^13^ and exerted no significant effects on hemodynamics.^68^ In some other animal studies, 5 cmH_2_O PEEP was considered to have more positive effect on oxygenation reduce shunt fraction and no further damage to cardiac function.^69^, ^70^, ^71^

### 3.3 5 to 10 cmH_2_O PEEP

PEEP levels of 5 to 10 cmH_2_O are assumed to be relatively safe, reliable, and tolerable. In clinical work, we often choose 5 to 10 cmH_2_O PEEP for one‐lung ventilation.

#### 3.3.1 Clinical research

The application of 5 to 10 cmH_2_O PEEP to the ventilated lung may ameliorate lung compliance, reduce the incidence of intraoperative hypoxemia,^72^, ^73^ and has only a minor effect on cardiac output and dead space fraction.^60^, ^74^, ^75^, ^76^, ^77^

Compared with PEEP ≤ 5 cmH_2_O, 5 to 10 cmH_2_O PEEP could significantly improve oxygenation and lung compliance during OLV and have the potential to reduce lung injury in patients, which may be related to the fact that a higher level of PEEP could maintain more lung unit expansion. Marret et al. found that 5 to 8 cmH_2_O PEEP can reduce the incidence of acute lung injury in patients undergoing lobectomy, shorten the length of hospital stay, and improve the prognosis of patients with lung cancer.^10^ Li H et al. have observed that applying 6 cmH_2_O PEEP did not increase intra ocular pressure but enhanced dynamic compliance and oxygenation during OLV and reduce the incidence of postoperative pulmonary complications such as atelectasis and pulmonary inflammatory lesions.^78^, ^79^ This may be due to the reduction of peak inspiratory pressure and plateau airway pressure during mechanical ventilation to balance the pressure between alveoli, so as to avoid alveolar collapse, increase air exchange, and reduce the effects of shear injury and inflammation, resulting in lung protection. In contrast, low‐level PEEP group (PEEP ≤ 5 cmH_2_O) and high‐level PEEP group (6 to 10 cmH_2_O PEEP) had no significant difference in the incidence of PPCs within 7 days after thoracoscopic pneumonectomy according to a randomized controlled clinical trial.^80^ Leong et al. randomized 46 patients receiving elective thoracotomy for lung resection into 0, 5, 8, or 10 cmH_2_O PEEP group. The primary endpoint was oxygenation and respiratory compliance of the dependent lung. They concluded that 5 to 10 cmH_2_O PEEP may not improve lung compliance and intraoperative or postoperative oxygenation.^81^ It is uncertain whether intraoperative one‐lung ventilation with 5 to 10 cmH_2_O PEEP is protective for thoracic surgical procedures. When it is selected, we must weigh improvements in pulmonary function against potential hemodynamic compromise and balance the possible benefits and risks.

#### 3.3.2 Animal research

In a porcine sick lung model, different levels of PEEP were used for one‐lung ventilation. It was found that moderate PEEP (5–10 cmH_2_O) helped to increase lung volume, improve oxygenation, and reduce intrapulmonary shunt.^13^ Conversely, Naik As et al. performed one‐lung ventilation in sheep and found that 7 cmH_2_O PEEP can lead to excessive alveolar dilation, damage pulmonary capillaries, and adversely affect hemodynamics.^39^ This may be due to the latter experiment being conducted in preterm lambs, and 7 cmH_2_O PEEP may cause more distension of alveoli and elevated neutrophils and expression of proinflammatory cytokines relative to low‐level PEEP.

### 3.4 10 cmH_2_O PEEP

When PEEP increased from zero to 10 cmH_2_O, the alveolar diameter increased in a positive proportion, while the thoracic pressure was almost constant. With the increase of the reopening alveolar, PaO_2_ and lung compliance may be affected to some extent.

#### 3.4.1 Clinical research

Based on previous findings, it suggested that lower levels of PEEP may not be sufficient to expand the collapsed alveoli and small airways. Some clinical studies have manifested that 10 cmH_2_O PEEP can prevent lung collapse and has smaller dead space, better oxygenation, and compliance than conventional recommended 5 cmH_2_O PEEP.^82^

Wolthuis Ek et al. found that, regardless of the left or right lying position, the respiratory compliance was the highest when PEEP was 10 cmH_2_O.^83^ Spadaro et al. reported in 41 patients undergoing video‐assisted thoracoscopic lobectomy or lung resection that 10 cmH_2_O PEEP may increase the oxygenation index and lower driving pressure without causing excessive alveolar expansion and increasing dead space compared with 5 cmH_2_O PEEP.^23^, ^64^ These scholars seemed to be in favor of the application of 10 cmH_2_O PEEP in clinical work.

Besides improving respiratory compliance and driving pressure, 10 cmH_2_O PEEP would lower inflammatory factors such as interleukin‐8 as reported by three studies.^60^, ^64^, ^84^ Wrigge et al. examined the effects of 10 cmH_2_O PEEP and ZEEP on intraoperative oxygenation and postoperative inflammatory mediator in patients undergoing thoracotomy. However, no difference was observed.^60^, ^85^, ^86^ Owing to increasing the pulmonary vascular resistance and redistributing the blood flow, 10 cmH_2_O PEEP may increase intrapulmonary shunt, thereby not conducive to the operation.^21^ In addition, it has potential hemodynamic hazards such as reducing cardiac output and pleural fluid composition^31^, ^87^ and increasing myocardial acceleration index.^52^, ^53^ It can be seen that 10 cmH_2_O PEEP should be applied with caution especially when patients have poor cardiac function or compensation. The benefits of 10 cmH_2_O PEEP are warranted to be further confirmed by prospective studies.

#### 3.4.2 Animal research

Sacramento EMF et al. applied 2, 5, and 10 cmH_2_O PEEP respectively to Sprague–Dawley male rats during lung transplantation. It was found that 10 cmH_2_O PEEP group was pertinent to improving oxygenation and hemodynamics as well as reducing the incidence of atelectasis of rats.^88^ The findings of Michelet et al. were consistent with it by analyzing the arterial blood gas of pigs at different levels of PEEP during one lung ventilation.^13^, ^89^ In addition, they found 10 cmH_2_O PEEP would reduce ventricular preload and cardiac output.

### 3.5 PEEP > 10 cmH_2_O

High‐level PEEP would cause alveolar hyperinflation, thus increasing lung parenchyma stress and strain and systemic organ injury.^90^ PEEP greater than 10 cmH_2_O is rarely used in clinical practice except at the beginning of a few operations to fully expand the alveoli.

#### 3.5.1 Clinical research

On one hand, high PEEP may correct hypoxemia and promote more homogenous ventilation by preventing atelectasis.^91^ On the other hand, it would indirectly contribute to circulatory depression by reducing venous/lymphatic drainage.^92^, ^93^, ^94^

In the study of Cylwik J, it was found that 91.9% of patients can successfully dilate the atelectasis area with an average PEEP of 17 cmH_2_O.^65^ It appears that atelectotrauma may be alleviated due to reducing the need for high concentration oxygen and the occurrence of ventilation‐perfusion mismatch.^14^, ^95^

Nevertheless, Jo et al. reported that PEEP greater than 11 cmH_2_O affected the stability of hemodynamics.^72^, ^96^ High‐level PEEP would lead to intrapulmonary pressure increased and the venous return blood volume and the cardiac output reduced,^97^, ^98^ which induced an reduction of the pulmonary blood perfusion and gas exchange.^96^ Some other studies demonstrated that there was a downward trend in lung compliance with the potential risk of barotrauma and bacterial translocation when PEEP value applied was excessively high.^53^, ^99^, ^100^, ^101^ In light of these limitations, PEEP greater than 10 cmH_2_O is rarely employed. It ought to be applied with a close control of cardiovascular hemodynamics, respiratory function and gas exchange.

#### 3.5.2 Animal research

One randomized controlled experiment comparing different levels of PEEP during one lung ventilation in a pig model showed that lung compliance decreased and oxygenation worsened significantly when PEEP greater than or equal to 15 cmH_2_O.^13^ Similar to the results of the study above, He Fang et al. found that PEEP exceeded 10 cmH_2_O would induced reducing lung compliance and increasing intrapulmonary shunt, thereby affecting the tissue oxygen supply.^102^

## 4 EXPLORE THE OPTIMAL INDIVIDUALIZED PEEP VALUE

The effect of PEEP is dual: on one hand, high‐level PEEP may lead to excessive alveolar expansion; on the other hand, the collapsed alveoli could not be fully opened if the PEEP value is too small. At the same time, the existence of individual heterogeneity in patients' physique, BMI, and physiological structure of lung makes the homogenized PEEP value not optimal for each individual patient. PEEP is then individually titrated to optimize lung compliance and minimize driving pressure when the elements above are taken into account.

Several targets have been proposed for individualized titration, including improving intraoperative oxygenation and hemodynamics, providing optimal pulmonary compliance,^28^, ^95^, ^103^, ^104^, ^105^, ^106^, ^107^, ^108^ and reducing the incidence of postoperative pulmonary complications.^24^, ^25^, ^26^, ^27^, ^109^, ^110^ An animal experiment also confirmed the importance of individualized PEEP, in which Reiniush et al. obtained the appropriate range of PEEP according to oxygenation, driving pressure and cardiac output. PEEP exceeded this range would cause ventilation/perfusion mismatch of the ventilated lung.^111^ The optimal PEEP remains a matter of considerable debate. This uncertainty has fostered various setting approaches. Some believe it should be titrated, whereas others advocate imaging methods. The following describes several methods.

### 4.1 Test titration

In clinical practice, PEEP titration could allow personalized application of optimal PEEP to achieve optimize lung function while matching the highest dynamic lung compliance, the best oxygenation index, and the minimum driving pressure.

#### 4.1.1 Dynamic compliance

Patients undergoing thoracic surgery often have potential differences in respiratory compliance due to the size or location of the mass or the existing lung disease.^109^ Dynamic pulmonary compliance (Cdyn) can reflect the number of ventilated alveoli. Maximum alveoli opening was attained at the PEEP associated with the highest Cdyn. When the PEEP value is lower than this level, the lungs begin to collapse. We hypothesized that a decremental PEEP trial might provide an appropriate means of PEEP thereby improving oxygenation and reducing right ventricular load^112^, ^113^ and the risk of ventilator‐induced lung injury.^46^ Several studies supported the hypothesis. Luecke T et al. found that compared with standardized 5 cmH_2_O, PEEP titration guided by Cdyn was shown to be more effective in improving oxygenation and respiratory mechanics.^114^ Xu D et al. compared different PEEP titration strategies and found it seemed to be inconsistent with the studies above in some way that the dynamic compliance method could lower driving pressure and improve dynamic compliance but does not adequately improve arterial oxygenation and intrapulmonary shunt.^115^ Suarez‐Sipmann et al. also demonstrated the role of dynamic lung compliance in setting the optimal PEEP value using animal models.^116^ The respiratory parameters displayed on anesthesia machine include pulmonary dynamic compliance. The method is simple, easy to apply, and offers great potential for larger prospective studies clearly support Cdyn‐guided PEEP during thoracic surgery.

#### 4.1.2 Pressure–volume curve

The pressure–volume curve (P‐V curve) reflects the compliance and resilience of the lung. PEEP above the low inflection point (LIP) of the P‐V curve was thought to recruit a large number of alveoli to protect lung from shear injury. Liu Wenjun et al. recommended that PEEP set at LIP + 2 cmH_2_O was optimal to reduce the traction stimulation and inflammatory response of lung tissue.^117^ Others hold different suggestions that PEEP corresponding to the inflection point on the expiratory branch of the P‐V curve was effective in meliorating lung function and arterial oxygenation and reducing the occurrence of acute lung injury.^118^, ^119^ The method is convenient and suitable in the operating room. Whether the P‐V curve can provide useful information about optimal PEEP is still in doubt. We are conducting a clinical trial comparing individualized PEEP based on P‐V curve with 5 cmH_2_O PEEP and 10 cmH_2_O PEEP. Our pre‐experimental results indicated individualized PEEP was associated with a downward trend of the incidence of postoperative pulmonary complications. It may add new information about the rational of using individualized PEEP during OLV.

#### 4.1.3 Intrinsic PEEP

Intrinsic PEEP (PEEPi) resulting from restriction to airflow in the bronchioles during exhalation reflect the residual pressure, which may reduce pulmonary dynamic compliance.^120^, ^121^, ^122^ In thoracic surgery, PEEPi can be exaggerated by lateral position, previous respiratory diseases, double lumen endotracheal intubation, and one lung ventilation. It may be necessary to add appropriate exogenous PEEP to offset PEEPi as well as avoid alveoli hyperdilatation.^29^ When exogenous PEEP is set to zero, expiration pauses and the measured end‐expiratory pressure value is PEEPi. PEEP should be set to 80% of PEEPi, which plays a certain role in reducing airway resistance and improving man–machine synchronization.

#### 4.1.4 Oxygenation index

There is a positive correlation between oxygenation index and lung compliance.^123^ The principle of the method is to determine optimal PEEP corresponding to the highest oxygenation index from blood gas analysis.^124^ However, it requires multiple arterial blood samples for blood gas analysis, which is expensive and cannot be monitored in real time, so it is not suitable for individualized PEEP titration during operation. Moreover, the PEEP value titrated by oxygenation index is comparatively high because it only focuses on the improvement of oxygenation but does not take ventilator‐induced lung injury into account.

#### 4.1.5 Driving pressure

Driving pressure, reflecting the change of alveolar pressure before and after inhalation, is defined as the ratio of tidal volume to respiratory compliance, which can be calculated by plateau airway pressure minus PEEP. Park et al. defined the PEEP value causing the minimum driving pressure as the optimal PEEP and found it permits reducing PPCs.^109^ The mechanism may be that lower driving pressure is pertinent to a higher survival rate and lower incidence of periodic strain.^125^, ^126^ While in a large multicenter randomized trial, PEEP guided by driving pressure did not reduce the incidence of PPCs though improving intraoperative lung compliance and arterial partial pressure of oxygen during pneumonectomy compared with 5 cmH_2_O PEEP.^127^ Wang F et al. believed that PEEP guided by driving pressure could effectively alleviate postoperative inflammatory response because the results showed the level of postoperative inflammatory factors of IL‐6 and HMGB1 were decreased, which may be related to its reducing the number of collapsed alveoli and maintaining the expansion of distal collapsed airway.^128^ The evidence supporting the driving pressure method to titrate individualized PEEP is limited. More clinical trials need to be carried out in the future.

#### 4.1.6 Dead space fraction

Dead space fraction refers to the ratio of dead space volume to tidal volume, reflecting collapse and re‐expansion of alveolar. As gas exchange is insufficient and the lungs are susceptible to VILI during OLV,^129^ monitoring dead space in real time plays an important role in adjusting ventilator settings.^130^ Given that the ideal PEEP minimizes dead space fraction to bring ventilation and blood flow into the best state, dead space fraction is an attractive physiological target for PEEP titration.^131^

#### 4.1.7 Transpulmonary pressure

Transpulmonary pressure is the pressure gradient from the airway to the pleural space, driving the contraction and expansion of alveoli. The minimum transpulmonary pressure, which can keep positive throughout the respiratory cycle to expand the collapsed alveoli, can be used as one of the selection criteria for PEEP titration. Esophageal manometry is one of the approaches to measuring transpulmonary pressure. Rauseo M et al. found that individualized PEEP titrated by esophageal manometry could significantly reduce the occurrence of intraoperative hypoxemia as well as improve pulmonary compliance and hemodynamics.^108^, ^132^ However, it is difficult to judge the best monitoring position by esophageal manometry due to a certain pressure gradient in the pleural cavity from the non‐ventilated lung to the ventilated lung in lateral position during thoracic surgery and heterogeneity in the lung tissue. The PEEP step method (PSM) determining the lung mechanics and chest wall mechanics of patients through the calculated change of end‐expiratory lung volume is another approach to measuring transpulmonary pressure and the measurement is highly consistent with esophageal manometry.^133^, ^134^, ^135^, ^136^ Although PSM is noninvasive, convenient, and can obtain the pressure in the whole chest cavity, it requires specialized monitoring equipment to record and cannot measure transpulmonary pressure continuously, which limit its adoption in clinical practice.

#### 4.1.8 Functional residual capacity

FRC is the volume of gas remaining in the lungs after calm exhalation, which can better reflect the alveolar recruitment and collapse compared with lung compliance.^137^ It can be used as a supplement to select the optimal PEEP. The FRC value was recorded at each PEEP level, and the last pressure level of PEEP corresponding to the lowest FRC was the optimal PEEP. Compared with oxygenation index and dynamic compliance method, the optimal PEEP guided by FRC benefits lung compliance, oxygenation index and hemodynamics while reducing oxidative stress response in patients undergoing radical lung cancer surgery.^138^ FRC method is not invasive, but it may lead to DO_2_ decreased, which should be comprehensively considered in clinical.

### 4.2 Imaging method

More precise indicators were called for to provide quantitative evaluation indicators for individualized PEEP such as computed tomography, electrical impedance tomography, and ultrasound.

#### 4.2.1 Computed tomography

Computed tomography (CT) can calculate the air content of lung tissue through the density, intuitively displaying the re‐expansion of collapsed alveoli.^139^, ^140^ It may be useful to adjust PEEP to the desired levels. However, it is unlikely that the CT approach to tailoring PEEP to individual patients is suitable in operation for its radiative and cumbersome and other techniques, which could be used at the bedside to reach similar information would replace it.

#### 4.2.2 Electrical impedance tomography

Electrical impedance tomography (EIT) is a bedside imaging method that visualizes local ventilation and lung perfusion distribution. Changes in CRS as well as alveolar overdistension and collapse can be assessed by EIT dynamically and noninvasively.^108^, ^141^, ^142^, ^143^, ^144^, ^145^, ^146^, ^147^, ^148^, ^149^, ^150^ Compared with 5 cmH_2_O PEEP, the individualized PEEP determined by EIT was effective in reducing the driving pressure and improving the survival rate and respiratory mechanics without affecting the hemodynamic stability.^14^, ^21^, ^44^, ^151^, ^152^, ^153^ In recent years, EIT has not yet been widely disseminated in thoracic surgery though it is popular in clinical trials as a method to select the optimal PEEP value. The reasons include but are not limited to its high price and only monitoring a single dimension of the thoracic cavity, which makes it difficult to reflect the overall ventilation of the lung.^154^ Moreover, electrodes placed in the 4th to 5th intercostal spaces at the parasternal line would affect the scope of surgical disinfection and maneuvers.^155^

#### 4.2.3 Lung ultrasonic score

Lung ultrasonic score (LUS) allowing non‐invasive monitoring of lung recruitment in real time has been proposed as a practical bedside imaging method alternative to CT method in the assessment of appropriate PEEP during OLV. The higher the LUS is, the more serious the atelectotrauma will be.^156^ The PEEP value when the LUS suddenly increases by more than 30% and then plus 2 cmH_2_O is defined as the best PEEP value. The individualized PEEP on the basis of LUS method could reduce the risk of hyperinflation of the ventilated lung with intraoperative oxygenation and pulmonary compliance improved. Ultrasound is a technique widely used in clinic whose advantage is its availability at the bedside and high‐cost performance. Nevertheless, the method has certain limitations in operation due to the ultrasonic probe placed on the chest wall with the lack of spatial resolution and the limited penetration of ultrasound.^157^

Individualized PEEP has been advocated to be applied in clinical though lacking solid evidence from evidence‐based medicine. A large number of studies above have shown the benefits of individualized PEEP to clinical outcomes. Conversely, Mascotto G et al. reported that it has no significant influence on the improvement of intraoperative and postoperative oxygenation.^28^, ^143^, ^158^

More recently, we mainly implement the low tidal volume ventilation mode involved in LPVS during thoracic surgery to ameliorate the respiratory and circulatory system. In addition to the low tidal volume ventilation mode, we gradually applied PEEP suitable for the individual characteristics of patients in clinical work. The beneficial effects of PEEP are pertinent to the prevention of atelectasis and avoiding the cyclical opening and collapse of alveoli.^159^ No guidelines exist on the best PEEP setting during OLV, which should be tailored to the patient in order to achieve beneficial consequences while limiting adverse reactions as much as possible. However, the concept of maximizing oxygenation and lung compliance and reducing the amount of dead space is commonly recognized. Therefore, further investigations are warranted to determine optimal PEEP to be implemented in routine clinical practice.

## 5 CONCLUSION

On the whole, PEEP has been widely accepted and applied in clinical as a part of LPVS. Different levels of PEEP have been used in thoracic surgery to improve oxygenation and reduce intrapulmonary shunt and postoperative pulmonary complications, but it is still necessary to explore a simple and economical method to determine the optimal PEEP value. Reaching an agreement on the determination represents a high priority of clinical trials and experiments.

## CONFLICT OF INTEREST

No potential conflict of interest was reported by all authors.

## AUTHOR CONTRIBUTION

**Jiang Yueyi**: Conceptualization (lead), writing–original draft (lead), formal analysis (lead), and writing–review and editing (equal). **Tan Jing**: Methodology (lead), writing–original draft (supporting), conceptualization (supporting), and writing–review and editing (equal). **Gu Lianbing**: Conceptualization (supporting), supervision (lead), and writing–review and editing (equal).

## Data Availability

Data sharing not applicable to this article as no datasets were generated or analyzed during the current study.
